# Impact of Ultra-Early Perioperative Antihypertensive Therapy in Acute Intracerebral Hemorrhage

**DOI:** 10.1161/STROKEAHA.125.053989

**Published:** 2026-03-24

**Authors:** Tao Liu, Linan Chen, Leibo Liu, Yang Liu, Lu Ma, Laurent Billot, Qiang Li, Zhihao Zhao, Alejandra Malavera, Paula Muñoz-Venturelli, Asita de Silva, Huy Thang Nguyen, Kolawole W. Wahab, Jeyaraj D. Pandian, Mohammad Wasay, Octavio M. Pontes-Neto, Rongcai Jiang, Carlos Abanto, Antonio Arauz, Lili Song, Chao You, Craig S. Anderson, Xin Hu, Xiaoying Chen

**Affiliations:** 16 George Institute for Global Health, Faculty of Medicine, University of New South Wales, Sydney, Australia (T.L., L.C., L.L., L.B., Q.L., A.M., P.M.-V., L.S., C.S.A., X.C.).; 1 Department of Neurosurgery, Tianjin Medical University General Hospital, China (Y.L., Z.Z.).; 2 Department of Neurosurgery, West China Hospital, Sichuan University, Chengdu, China (L.M., C.Y., X.H.).; 3 Clinical Research Center, Faculty of Medicine Clinica Alemana Universidad del Desarrollo, Santiago, Chile (P.M.-V., C.S.A.).; 4 Clinical Trials Unit, Faculty of Medicine, University of Kelaniya, Colombo, Sri Lanka (A.d.S.).; 5 Stroke Unit, 115 Hospital, Ho Chi Minh City, Vietnam (H.T.N.).; 6 Department of Medicine, University of Ilorin & University of Ilorin Teaching Hospital, Nigeria (K.W.W.).; 7 Neurology Department, Christian Medical College and Hospital, Ludhiana, India (J.D.P.).; 8 Department of Medicine, The Aga Khan University, Karachi, Pakistan (M.W.).; 9 Department of Neuroscience and Behavioral Sciences, Ribeirão Preto Medical School, University of São Paulo, Brazil (O.M.P.-N.).; 10 The Cerebrovascular Disease Research Center, National Institute of Neurological Sciences, Lima, Peru (C.A.).; 11 Instituto Nacional de Neurologia y Neurocirugia Manuel Velasco Suarez, Mexico City, Mexico (A.A.).; 12 Institute of Science and Technology for Brain-inspired Intelligence, Fudan University, Shanghai, China (L.S., C.S.A.).; 13 Key Laboratory of Computational Neuroscience and Brain-Inspired Intelligence (Fudan University), Ministry of Education, Shanghai, China (L.S., C.S.A.).; 14 Department of Neurology, Royal Prince Alfred Hospital, Sydney, Australia (C.S.A.).; 15 The George Institute for Global Health China, Beijing, China (C.S.A.).

**Keywords:** blood pressure, cerebral hemorrhage, Glasgow Coma Scale, hematoma, quality of life

## Abstract

**BACKGROUND:**

Early intensive blood pressure (BP) lowering improves outcomes in acute intracerebral hemorrhage, but its perioperative benefit among patients undergoing surgical hematoma evacuation is uncertain. We evaluated whether earlier achievement of intensive BP targets is associated with improved outcomes in this population.

**METHODS:**

Post hoc secondary analysis of the INTERACT3 (the third Intensive Care Bundle With Blood Pressure Reduction in Acute Cerebral Haemorrhage Trial) pragmatic, international, multicenter, blinded-end point, and stepped-wedge cluster-randomized trial. Among 7036 enrolled intracerebral hemorrhage patients at 121 hospitals, those who underwent surgical hematoma evacuation were included. Patients were categorized by time from hospital arrival to achieving the target systolic BP <140 mm Hg: ≤2 hours versus >2 hours. The primary outcome was 6-month mortality. Key secondary outcomes included death or disability (modified Rankin Scale scores 4–6), modified Rankin Scale score shift, health-related quality-of-life (EuroQol 5-Dimension 3-Level [EQ-5D-3L] domains, visual analog scale, and health utility index), and serious adverse events. Adjusted associations were estimated using Cox, logistic, ordinal logistic, and linear regression models, controlling for age, sex, treatment type, and admission Glasgow Coma Scale.

**RESULTS:**

Of 7036 patients with acute intracerebral hemorrhage, 1506 underwent surgical hematoma evacuation (mean [SD] age, 59.7 [11.8] years; 33.9% women). Overall, there was no statistically significant difference in 6-month mortality between patients who achieved target BP within 2 hours of treatment initiation and those who achieved it after 2 hours (adjusted hazard ratio, 0.81 [95% CI, 0.63–1.04]; *P*=0.09). Early BP achievement was associated with a lower risk of death or disability (adjusted odds ratio [OR], 0.71 [95% CI, 0.56–0.90]; *P*=0.01), a favorable shift in the distribution of modified Rankin Scale scores (adjusted common OR, 0.73 [95% CI, 0.60–0.89]; *P*<0.01), and fewer serious adverse events (adjusted OR, 0.73 [95% CI, 0.57–0.94]; *P*=0.02). EuroQol 5-Dimension 3-Level outcomes also favored the early group, with significant improvements in mobility (adjusted OR, 0.76 [95% CI, 0.60–0.97]; *P*=0.03), pain/discomfort (adjusted OR, 0.72 [95% CI, 0.54–0.95]; *P*=0.02), and usual activities (adjusted OR, 0.79 [95% CI, 0.62–1.00]; *P*=0.05), as well as higher visual analog scale (mean difference, 0.08 [95% CI, 0.002–0.17]; *P*=0.04) and health utility scores (mean difference, 0.05 [95% CI, 0.02–0.09]; *P*<0.01).

**CONCLUSIONS:**

In patients with intracerebral hemorrhage undergoing surgical hematoma evacuation, perioperative intensive BP reduction appears safe. Achieving systolic BP <140 mm Hg within 2 hours was associated with better functional and quality-of-life outcomes, and fewer serious adverse events. These time-sensitive associations support prioritizing ultra-early perioperative BP control; confirmatory prospective analyses are warranted.

**REGISTRATION:**

URL: https://www.clinicaltrials.gov; Unique identifier: NCT03209258.

Intracerebral hemorrhage (ICH) is among the most lethal subtypes of stroke, with ≈40% of patients dying within the first month and most survivors experiencing significant long-term disability.^1^ A marked acute elevation in blood pressure (BP) commonly occurs after ICH onset and is strongly associated with hematoma expansion. Therefore, early intensive BP reduction has emerged as a critical therapeutic strategy to limit hematoma growth and enhance patient outcomes.^2^ Recent evidence suggests that combining BP control with additional physiological interventions as part of a protocol-driven care bundle may confer additional clinical benefits.^3^

The third Intensive Care Bundle (INTERACT3 [the third Intensive Care Bundle With Blood Pressure Reduction in Acute Cerebral Haemorrhage Trial]) is a large, international, pragmatic clinical trial designed to assess whether managing multiple critical physiological parameters in a hospital setting, including early intensive BP lowering, glucose and temperature control, and anticoagulant reversal, can improve functional outcomes for patients with acute ICH.^3^ The INTERACT3 demonstrated that implementing a comprehensive care strategy within hours of symptom onset can enhance outcomes for these patients. Secondary outcomes assessed included mortality, serious adverse events (SAEs), health-related quality of life (measured by the EuroQol 5-Dimension 3-Level [EQ-5D-3L] self-report questionnaire), and patient residence (own home versus other). This approach emphasizes synchronized optimization of multiple parameters, offering a multidimensional, patient-centered standard of care for patients with ICH, particularly in resource-limited low- and middle-income countries.

A notable aspect of INTERACT3 is that participants in both groups underwent hematoma evacuation at similar rates (26.4% versus 26.9%), though the time taken to reach the target systolic BP varied significantly (median, 2.3 hours, interquartile range [IQR] 0.8–8.0 hours versus median 4.0 hours [IQR, 1.9–16.0 hours]).^3^ To date, limited clinical research exists on intensive BP management during perioperative management. Available evidence suggests that while intensive BP reduction during the perioperative period is generally safe, its impact on improving clinical outcomes or reducing rebleeding is limited.^4^ Theoretically, the association of perioperative BP and AEs could follow a U-shaped relationship, a hypothesis recently supported by research indicating the lowest risk of postoperative AEs at a preoperative SBP of 143 mm Hg.^5^ A meta-analysis of randomized controlled trials further supports that targeting lower BP can reduce mortality, atrial fibrillation, and the need for transfusions.^6^ Overall, intensive BP management is considered safe for perioperative patients.

Despite the implementation of strict emergency BP reduction protocols, some patients in clinical practice still fail to reach target BP levels. The main factors contributing to this are the initial BP level and severity of disease, although these patients receive higher frequencies and larger doses of antihypertensive medication.^7^ A secondary analysis of the ATACH-2 trial (The Antihypertensive Treatment of Acute Cerebral Hemorrhage II) found that early intensive BP reduction (within 2 hours of symptom onset) was associated with reduced hematoma volume and improved outcomes.^8^ Similarly, the recently published intensive ambulance-delivered BP reduction (INTERACT4 study [Intensive Ambulance-Delivered Blood-Pressure Reduction in Hyperacute Stroke]) demonstrated that initiating intensive BP management within 2 hours of symptom onset in prehospital settings was linked to a lower incidence of functional impairment in hemorrhagic stroke patients.^9^ However, existing research has not specifically investigated the efficacy and safety of ultra-early intensive BP reduction in perioperative patients with acute ICH.

Because nearly half of the patients in the INTERACT3 trial reached the target systolic BP within 2 hours of treatment, this study offers a unique opportunity to explore this management approach further. Consequently, we conducted a perioperative subgroup analysis of INTERACT3 to identify the association between achieving target BP within 2 hours (early) versus after 2 hours (delayed) and patient outcomes.

## Methods

### Data Availability

Individual, deidentified participant data used in these analyses can be shared on request from any qualified investigator after approval of a protocol and signing of a data access agreement via both the trial steering committee and the Research Office of The George Institute for Global Health, Sydney, Australia.

### Data Sources and Setting

The INTERACT3 trial, an international randomized clinical study, has had its rationale, design, and main results previously published.^3,10^ This trial was conducted as a prospective, multicenter, stepped-wedge, cluster-randomized, controlled study across 121 hospitals in 9 low- and middle-income countries (Brazil, China, India, Mexico, Nigeria, Pakistan, Peru, Sri Lanka, and Vietnam) and 1 high-income country (Chile). Briefly, the INTERACT3 was done to implement a goal-directed care bundle incorporating intensive interventions for managing hypertension, hyperglycemia, pyrexia, and abnormal anticoagulation in patients with acute spontaneous ICH. The INTERACT3 trial protocol, along with written informed consent, received approval from the ethics committees of the participating hospitals and relevant regulatory authorities. For this secondary analysis, further ethical approval was not required as the data had already been deidentified. We report this secondary analysis in accordance with the STROBE guidelines (Strengthening the Reporting of Observational Studies in Epidemiology), with the completed checklist provided in the Supplemental Material.

### Study Population

Eligible participants in the INTERACT3 trial were adults, both male and female, aged 18 years or older, with imaging-confirmed spontaneous ICH within 6 hours of symptom onset. For this secondary analysis, only those who underwent surgical hematoma evacuation and achieved a target systolic BP of <140 mm Hg during the perioperative period were included. Patients who did not reach the target within 72 hours of the initiation of treatment were excluded from the analysis. All other participants meeting the above criteria were included in this post hoc analysis.

### Data Collection

To maintain consistent recruitment, all participating centers registered patients diagnosed with ICH. Baseline demographic and clinical data were collected on admission for enrolled patients. Follow-up data on clinical status, management, outcomes on day 1 and day 7 of treatment (or earlier in cases of discharge or death), and outcomes at 6 months were gathered by subcenter researchers and assessed by independent staff. Detailed information on these procedures has been previously published for the INTERACT3 trial.^3,10^

### Outcome Measures

In this secondary analysis, the primary outcome was mortality at 6 months. The secondary outcomes included death or disability (modified Rankin Scale [mRS] score 4–6), major disability in survivors (mRS score 3–5), mRS score shift, hematoma expansion (defined as >33% relative or >6 mL absolute volume increase at 24 hours compared with baseline),^11,12^ neurological deterioration (defined as an increase of ≥4 points in the National Institutes of Health Stroke Scale score at day 7 compared with baseline), health-related quality of life (EQ-5D-3L self-report questionnaire), residence at home, and all-cause SAEs. Baseline and Day 1 (24-hour) hematoma volumes were obtained from electronic case report forms as reported by participating sites based on routine clinical imaging assessment. SAEs were recorded throughout the follow-up period and were defined as events that resulted in death, were life-threatening, required inpatient hospitalization or prolongation of an existing hospitalization, resulted in persistent or significant disability or incapacity, involved a congenital anomaly or birth defect, or were considered other medically important events; a patient could experience >1 SAE.

### Statistical Analysis

Baseline characteristics were categorized based on the earlier time (≤2 hours) versus the delayed time (>2 hours) to reaching the targeted BP. Normally distributed continuous variables were presented as mean (SD), while non-normally distributed ones were shown as median (IQR). Categorical variables were displayed as frequencies with percentages (%). Group comparisons were made using t-tests for normally distributed continuous variables, Wilcoxon rank-sum tests for non-normally distributed variables, and χ^2^ or Fisher exact tests for binary variables. Trends in ordinal categorical variables were assessed using the Cochran-Armitage test.

A Cox regression model was used to assess the risk of mortality at 6 months, providing hazard ratios with 95% CIs, adjusting for a parsimonious set of key baseline prognostic covariates: age, sex, treatment type (care bundle versus usual care), and baseline Glasgow Coma Scale. To illustrate differences in mortality between the groups, cumulative mortality curves at 6 months were plotted.

The association (mean difference, 95% CI) between groups and visual analog scale (VAS) or utility score (measured by EQ-5D-3L) was examined using a linear regression model, with adjustments for the same parsimonious set of key baseline prognostic covariates (age, sex, treatment type, and baseline Glasgow Coma Scale). An ordinal logistic regression model was used to explore the shift in the distribution of mRS scores (mRS score shift). The 5 dimensions of health-related quality of life—mobility, self-care, usual activities, pain/discomfort, and anxiety/depression—were also analyzed using similar methods. A logistic regression model was used to evaluate the association (odds ratio [OR], 95% CI) between groups and the risk of other secondary outcomes, adjusting for the same variables.

To determine whether age, sex and treatment type affected the results, we examined the impact of earlier time (≤2 hours) versus delayed time (>2 hours) on the primary and secondary outcomes by age (<60 years (median age) and ≥60 years), sex (male and female), and treatment group (care bundle and usual care). Furthermore, additional exploratory subgroup analyses were performed to assess potential effect modification by ICH location (cortical, deep, or brainstem/cerebellar) and surgery type (craniotomy versus minimally invasive surgery). Interaction terms were included in the adjusted models, and *P* for interaction was reported.

Sensitivity analyses were performed with additional adjustment for premorbid functional status (preonset mRS score), baseline BP (systolic and diastolic), baseline blood glucose, baseline hematoma volume, and the presence of intraventricular hemorrhage across the primary and secondary outcomes. All models were fitted using a complete-case approach, including only participants with nonmissing data for the exposure, outcome, and covariates included in that model.

All analyses were conducted using R version 3.4.2 (R Project for Statistical Computing). All hypothesis tests were 2-sided, with a *P* value of <0.05 considered statistically significant.

## Results

Between December 12, 2017, and December 31, 2021, a total of 10 857 patients with acute ICH were screened for eligibility, and 7036 were included in the final INTERACT3 analysis. Among them, 1860 required surgical hematoma evacuation. However, 354 patients were excluded for not reaching the target within 72 hours, leaving 1506 patients for this secondary analysis. Of these, 618 achieved the target BP within 2 hours, while 888 took longer (Figure).

**Figure.**
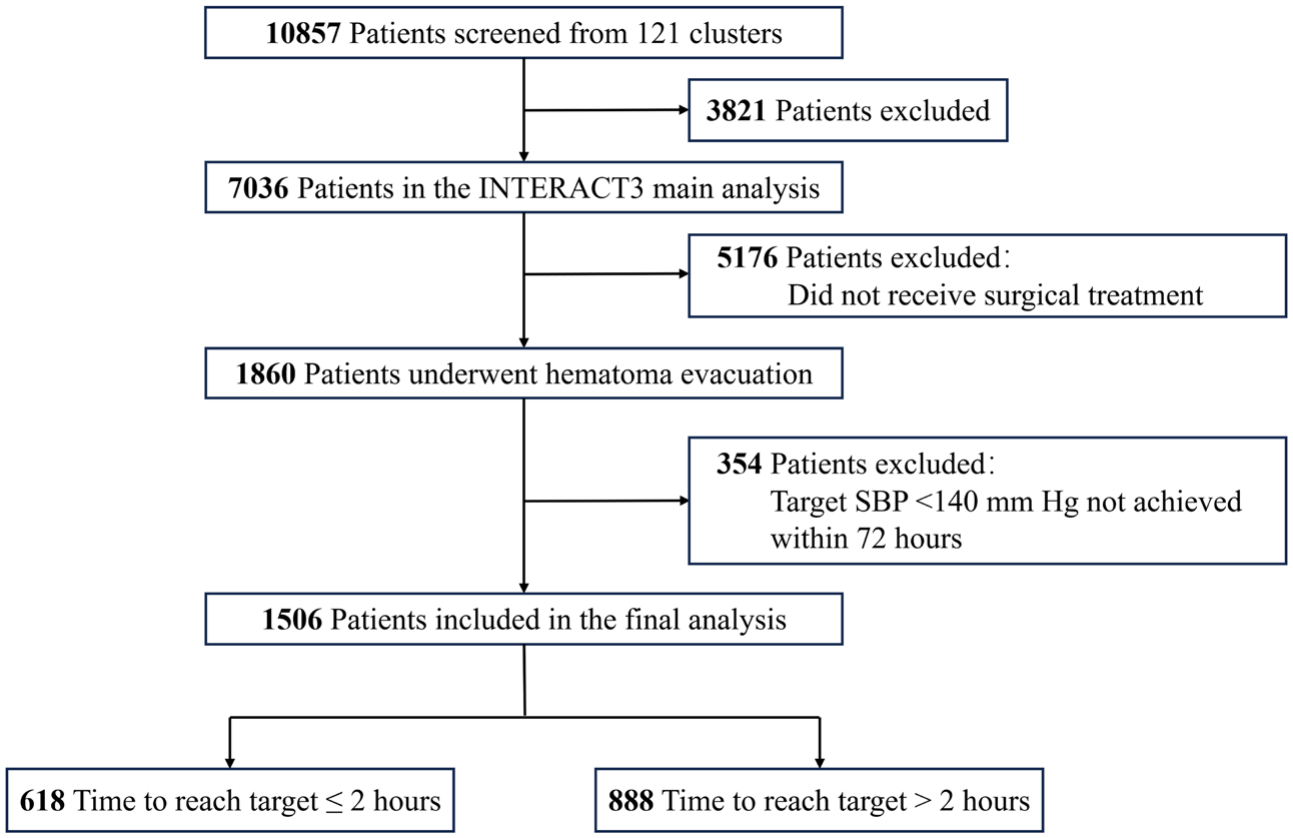
**Study flowchart.** INTERACT3 indicates the third Intensive Care Bundle With Blood Pressure Reduction in Acute Cerebral Haemorrhage Trial; and SBP, systolic blood pressure.

Included participants had a mean (SD) age of 59.7 (11.8) years, with 511 (33.9%) being female. On admission, the mean (SD) systolic BP was 180.5 (26.1) mm Hg, and 714 patients (47.4%) received care bundle interventions. Of the participants, 624 (41.43%) had a history of antihypertensive medication use, 344 (22.84%) were smokers, and 342 (22.71%) consumed alcohol. Overall, the median (IQR) hematoma volume was 35.0 (20.0–50.0) mL, and 677 patients (45.0%) had intraventricular hemorrhage. Further baseline characteristics are detailed in Table 1.

**Table 1.**
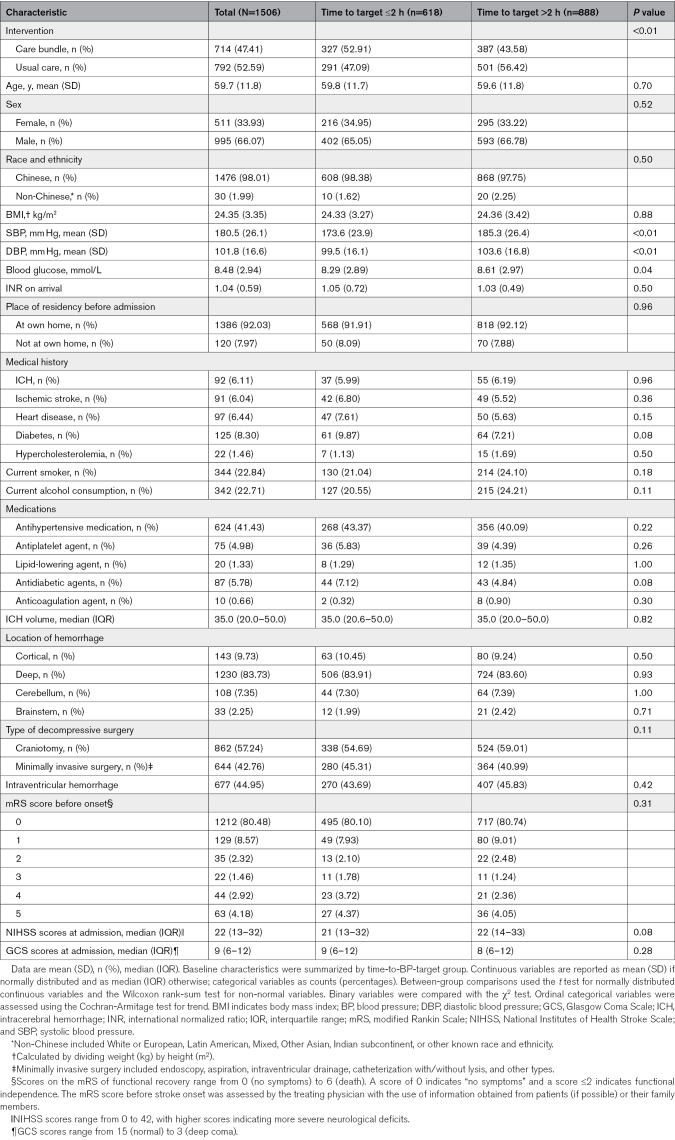
Baseline Characteristics of 1506 Patients Undergoing Surgical Hematoma Evacuation Grouped by Time to Reaching Target

Outcomes of comparison between the 2 groups are summarized in Table 2. Participants who achieved the target BP within 2 hours had a median (IQR) National Institutes of Health Stroke Scale score of 16 (10–28) at 7 days, compared with 18 (10–30) for those who reached the target beyond 2 hours. At 6 months, all-cause mortality was 18.52% (95% CI, 15.33–22.06) in the ≤2 hour group compared with 23.39% (95% CI, 20.42–26.56) in the >2 hours group. The incidence of death or major disability was 51.67% (95% CI, 47.36–55.96) versus 60.45% (95% CI, 56.87–63.94), respectively.

**Table 2.**
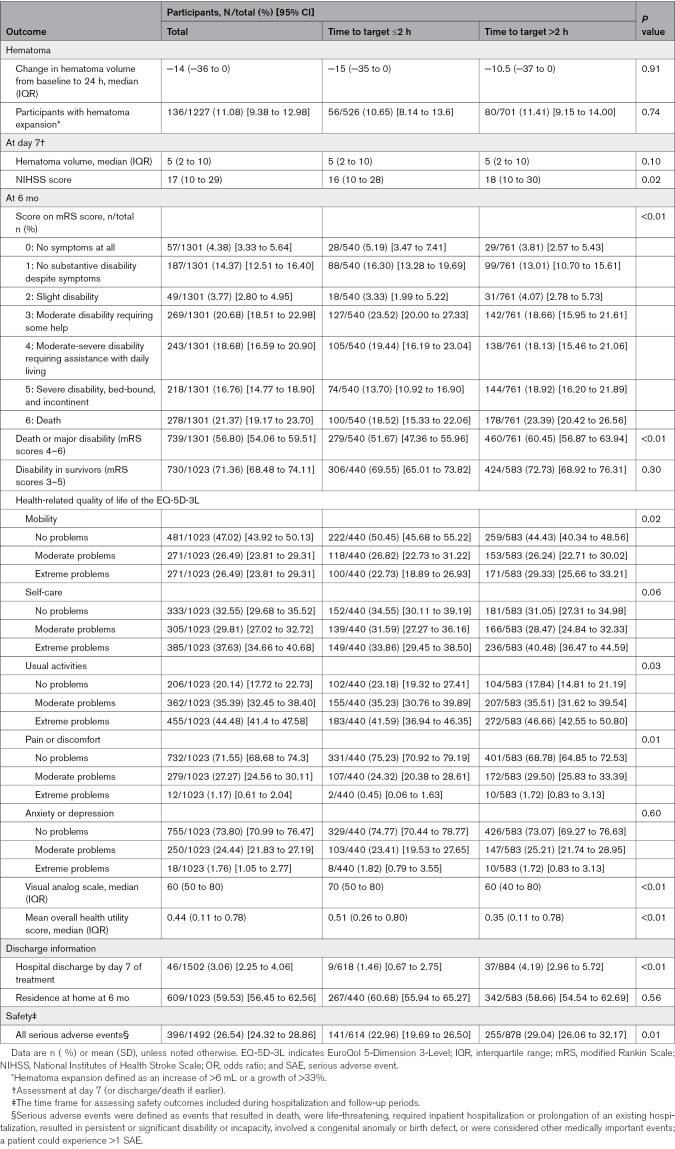
Outcomes of Comparison According to Time to Reaching Target

Participants who reached target BP within 2 hours also showed higher scores on the VAS and health utility scores, with VAS scores of 70 (IQR, 50–80) versus 60 (IQR, 40–80), and health utility scores of 0.51 (IQR, 0.26–0.80) versus 0.35 (IQR, 0.11–0.78) compared with those who reached the target later. SAEs occurred in 22.96 per 100 participants (95% CI, 19.69–26.50) among those who reached the target within 2 hours, compared with 29.04 per 100 participants (95% CI, 26.06–32.17) for those with a delay.

In analyses adjusted for age, sex, treatment type, and baseline Glasgow Coma Scale scores, patients who reached the target BP within 2 hours had a more favorable distribution of mRS scores at 6 months than those who took longer (adjusted common OR, 0.73 [95% CI, 0.60–0.89]; *P*<0.01; Table 3). Overall, the cumulative mortality did not differ significantly between the ≤2 hours group and the >2 hours group (adjusted hazard ratio, 0.81 [95% CI, 0.63–1.04]; *P*=0.09; Figure S1; Table 3). In the adjusted model, patients who reached target BP within 2 hours had a 29% lower risk of death or major disability (adjusted OR, 0.71 [95% CI, 0.56–0.90]; *P*=0.01) and a 27% lower risk of experiencing SAEs (adjusted OR, 0.73 [95% CI, 0.57–0.94]; *P*=0.02) compared with those who took longer. Similarly, earlier target achievement was associated with a lower risk of neurological deterioration (adjusted OR, 0.61 [95% CI, 0.43–0.88]; *P*<0.01). However, no significant differences were observed between the 2 groups regarding hematoma expansion (adjusted OR, 0.93 [95% CI, 0.65–1.34]; *P*=0.70) or residence at home at 6 months (adjusted OR, 1.07 [95% CI, 0.83–1.39]; *P*=0.58).

**Table 3.**
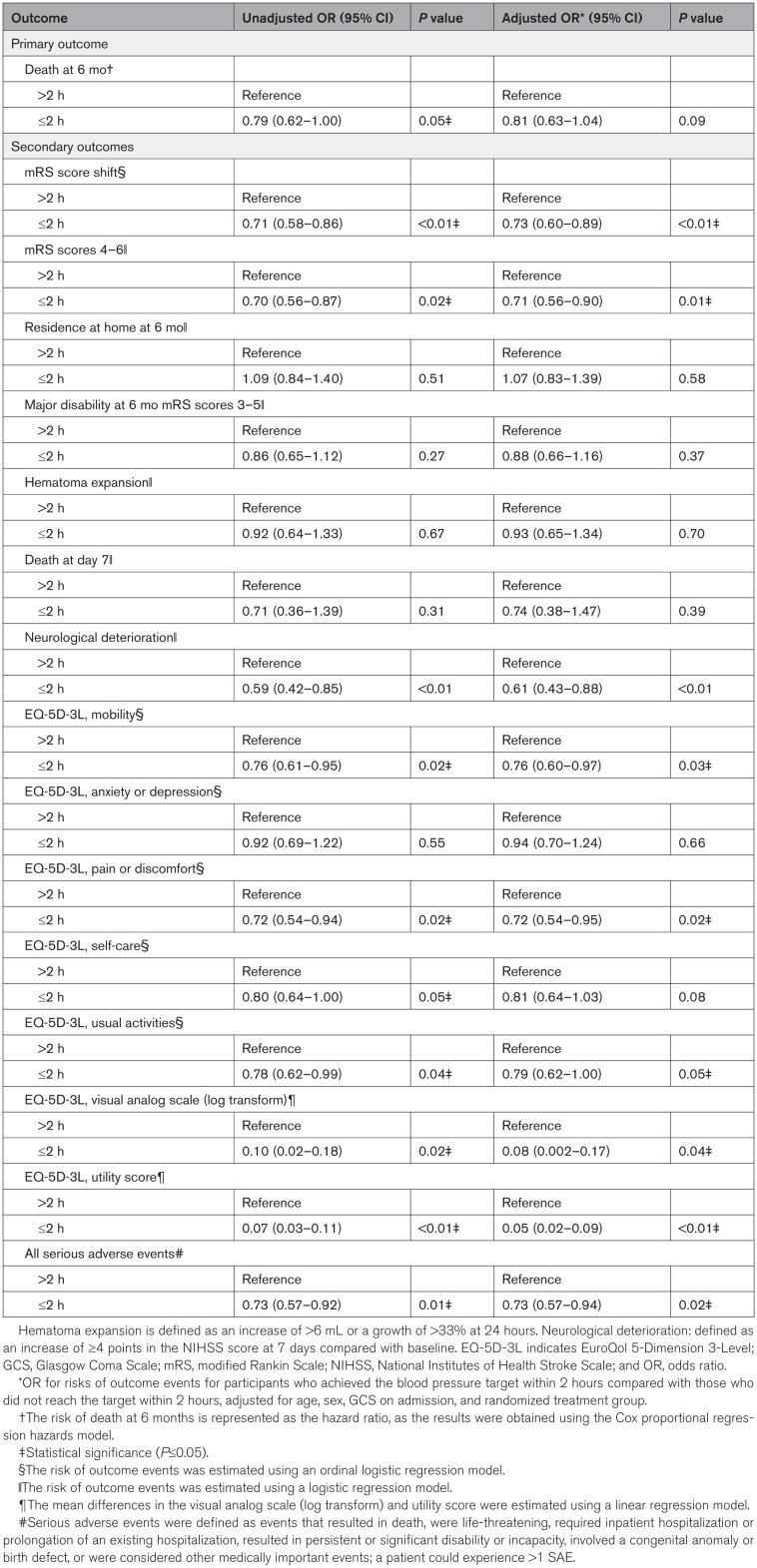
Multivariable Models and Risk of Outcomes in Time to Reaching Target

Additionally, the ≤2 hour group showed better outcomes in EQ-5D-3L measures, including mobility (adjusted OR, 0.76 [95% CI, 0.60–0.97]; *P*=0.03), pain or discomfort (adjusted OR, 0.72 [95% CI, 0.54–0.95]; *P*=0.02), and usual activities (adjusted OR, 0.79 [95% CI, 0.62–1.00]; *P*=0.05). Participants in the ≤2 hour group also had improvements in VAS and utility scores, which increased by 0.08 (95% CI, 0.002–0.17, *P*=0.04) and 0.05 (95% CI, 0.02–0.09, *P*<0.01), respectively, compared with those in the >2 hours group.

The subgroup analysis compared the effects of time to achieving target BP (≤2 versus >2 hours [reference]) on patient outcomes. The results suggest that the effects of time to achieving target BP on patient outcomes vary based on age, sex, and treatment type (care bundle versus usual care). Details are provided in Table 4. In additional exploratory subgroup analyses, generally consistent associations were observed for the primary and key secondary outcomes across hemorrhage location (cortical, deep, or brainstem/cerebellar) and surgery type (craniotomy versus minimally invasive surgery; Table S1). An isolated interaction was noted only for the EQ-5D-3L anxiety/depression domain by surgery type (*P* for interaction=0.04), with no other significant interactions observed.

**Table 4.**
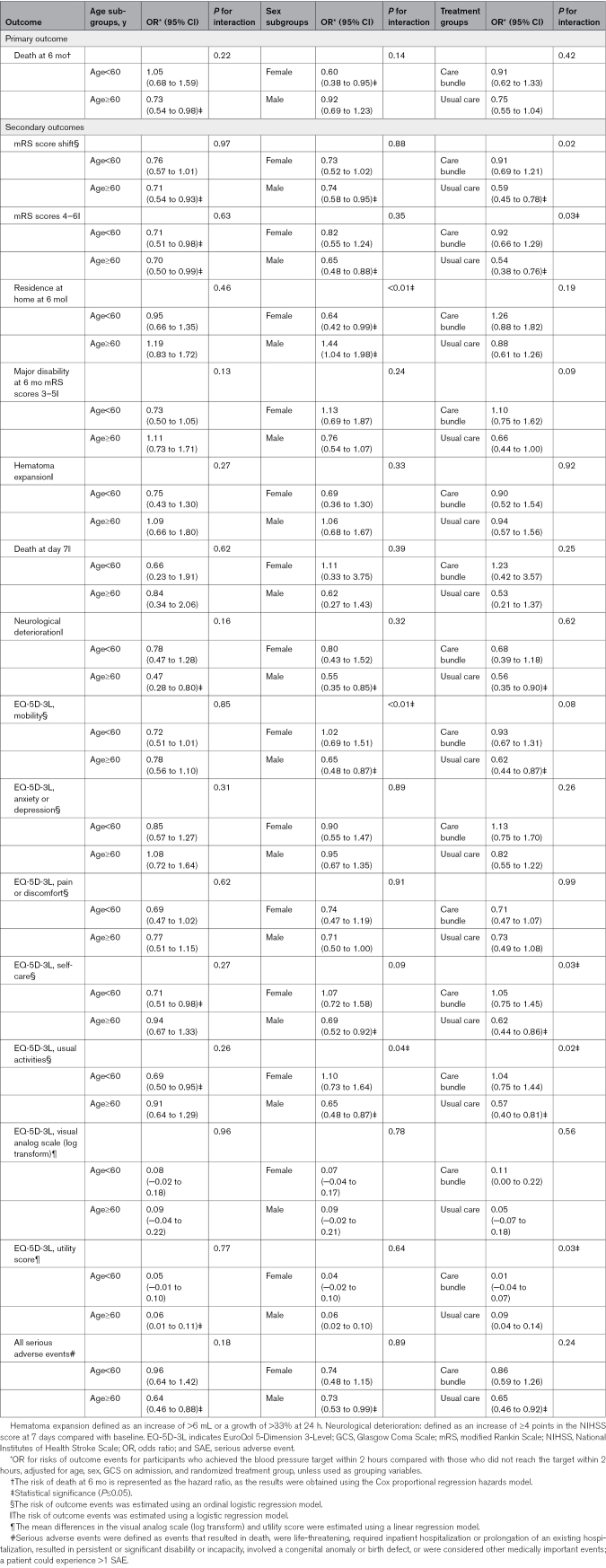
Subgroup Analysis

Sensitivity analyses with additional adjustment for premorbid functional status (preonset mRS score), baseline BP (systolic and diastolic), baseline blood glucose, baseline hematoma volume, and the presence of intraventricular hemorrhage showed no material changes in effect estimates or conclusions (Table S2).

## Discussion

The INTERACT3 trial provides the most extensive data set to date on perioperative intensive BP reduction in patients with acute ICH. In this post hoc analysis, we found that the clinical benefits of intensive BP management among patients undergoing hematoma evacuation were strongly time-dependent. Achieving the target BP within 2 hours, compared with delayed control (>2 hours), was associated with better functional and quality-of-life outcomes, and fewer SAEs. These findings suggest that ultra-early BP reduction in the perioperative setting is both feasible and safe, potentially improving long-term functional recovery.

There was no statistically significant difference in the primary outcome of 6-month mortality between the groups. However, patients who achieved target BP within 2 hours showed some other better clinical outcomes, particularly in mRS score shifts and rates of death or disability at 6 months. Furthermore, EQ-5D-3L analysis revealed that early BP control was associated with higher quality-of-life scores, particularly in domains such as mobility, pain or discomfort, and usual activities. These patients also had better VAS and health utility scores, aligning with findings from the pooled analysis of INTERACT studies, which support the benefits of early BP control on long-term quality of life.^13^

BP management is a crucial aspect of ICH treatment. Epidemiological studies indicate that over half of patients with ICH experience hypertension.^14,15^ Compared with patients with other causes of ICH, those with hypertension-induced ICH exhibit persistently higher systolic BP within the first 72 hours after admission.^16^ The hypertensive state observed in patients with ICH is driven by multiple factors and may be independent of catecholamine levels, which adds complexity to its management.^17^ The PROGRESS trial (Perindopril Protection Against Recurrent Stroke Study) in 2004 first demonstrated that BP management could reduce the relative risk of stroke recurrence by 50% among hemorrhagic stroke survivors, prompting advancements in BP management standards.^18,19^ Real-world data further support the protective effect of antihypertensive treatment on recurrence risk in patients with ICH. Over the past 40 years, recurrence rates have declined from 10.3 cases per 100 person-years during the OCSP period to 3.1 cases per 100 person-years during the OXVASC (Oxford Vascular Study) period. This reduction is especially notable in patients under 75 years of age, in whom the 5-year stroke recurrence risk decreased from 35.4% to 6.9%.^20^

Elevated BP is a risk factor for stroke recurrence and cognitive decline in patients with ICH, making early and aggressive BP reduction crucial for improving outcomes, particularly in cases of lobar hemorrhage.^21–23^ Animal models have shown that although intensive BP reduction does not significantly impact the blood-brain barrier in acute ICH, it effectively reduces hematoma expansion and improves neurological function in the early stages of hemorrhage.^24^ Multiple clinical studies further indicate that early intensive BP management is especially critical for limiting hematoma growth, particularly in patients with rapid hemorrhage expansion, which can improve functional outcomes.^25–29^ However, whether intensive BP reduction confers benefits to patients with ICH remains unproven.

Preoperative hypertension can lead to intraoperative hemodynamic instability, thereby increasing the risk of cardiovascular and cerebrovascular complications during the perioperative period.^30,31^ Retrospective studies have shown a significant association between perioperative hypertension and the incidence of postoperative ICH in craniotomy patients.^32^ A Bayesian meta-analysis suggests that, compared with higher BP targets, a lower BP target in the perioperative period may reduce mortality by 10% and decrease the incidence of atrial fibrillation as well as the need for transfusions.^5^ However, a small randomized controlled trial in 2017 found that intensive BP reduction in perioperative patients with ICH, targeting systolic BP between 120 and 140 mm Hg, did not significantly reduce rates of rebleeding, mortality, or other SAEs.^4^ Although this randomized controlled trial did not demonstrate clear benefits of intensive BP reduction, it suggests that perioperative BP control is safe.

Beyond the small sample size, the timing of intervention is a key factor influencing outcomes. A post hoc exploratory analysis of ATACH-2 suggests that ultra-early intensive BP reduction (within 2 hours of symptom onset) significantly reduces hematoma expansion, enhances functional independence, and improves overall prognosis.^8^ Previous studies indicate that early brain injury factors, such as hematoma expansion, emerge within hours of onset and worsen outcomes over time.^33,34^ Secondary analysis of INTERACT2 further supports this, showing a 22% reduction in hematoma growth when intensive BP management begins within 2.9 hours of onset, compared with only a 3% reduction when initiated after 4.9 hours.^35^ However, in this analysis, no significant difference in hematoma expansion was found between the early and delayed intervention groups. We think that for patients with ICH requiring surgery, who typically present with complex, severe conditions and rapid neurological decline, focusing solely on short-term outcomes, such as hematoma expansion, may be inappropriate. Greater emphasis should be placed on improving long-term survival and overall prognosis.

This study holds several important clinical implications. Although early intensive BP reduction is the standard of care for patients with spontaneous ICH, its application in the perioperative setting is often complicated by concerns about hemodynamic instability related to anesthesia and surgical stress. Our findings help bridge this evidence gap by showing that ultra-early achievement of the target systolic BP <140 mm Hg within 2 hours of treatment initiation is safe and feasible even in this high-risk population. This reinforces the concept that decisions regarding surgical hematoma evacuation should not delay the initiation of intensive BP control. By demonstrating that ultra-early intervention was associated with improved health-related quality of life and fewer SAEs, our data support prioritizing immediate BP optimization as an integral bridge to surgery, rather than viewing it as a separate or secondary consideration in the care pathway. Time to BP target may therefore be considered a pragmatic process-of-care indicator for quality improvement in the perioperative management of ICH.

However, this study also has limitations. As a post hoc analysis, this study is exploratory in nature. We did not apply formal statistical corrections for multiple comparisons across secondary outcomes, which may increase the risk of type I error; therefore, these findings should be interpreted with caution and require validation in high-quality randomized controlled trials. Further, the surgical and nursing standards across participating hospitals were not quantifiable, although such factors are critical to patient outcomes and currently lack standardized assessment methods. Despite adjusting for key confounders, there may still be unmeasured confounding factors. In addition, we did not impute missing data; complete-case analyses may reduce precision and could introduce bias if data are not missing at random. Lastly, due to data recording limitations, it is unclear whether the target BP was reached preoperatively, intraoperatively, or postoperatively, preventing further comparisons based on timing.

## Conclusions

In our post hoc analysis of the INTERACT3 trial, we found that perioperative patients with ICH who achieved target systolic BP within 2 hours of initial treatment had better functional and quality-of-life outcomes and fewer SAEs. Ultra-early intensive BP reduction in the perioperative period appears both safe and feasible.

## Article Information

### Acknowledgments

The authors also thank the participants, their relatives, and their families.

### Disclosures

Dr Muñoz Venturelli reports grants from Boehringer Ingelheim, ANID, and Pfizer. Dr Wahab reports service as Editor for the Nigerian Society of Neurological Sciences; service as Member, Guidelines Review Committee for the World Stroke Organization; and service as Secretary-General for the Nigerian Hypertension Society. Dr Pontes-Neto reports compensation from Medtronic, Servier Pharmaceuticals LLC, Bayer, and Pfizer for other services and compensation from Boehringer Ingelheim for consultant services. Dr Anderson is a senior investigator fellow for the National Health and Medical Research Council of Australia. Dr Anderson reports grants from the National Health and Medical Research Council; compensation from AstraZeneca Australia and Auzone Pharma, China, for consultant services. The other authors report no conflicts.

### Supplemental Material

Tables S1–S2

Figure S1

STROBE Checklist

## Supplementary Material
