# Supplementary material for: Impact of Ultra-Early Perioperative Antihypertensive Therapy in Acute Intracerebral Hemorrhage
Source: Stroke. 2026 Mar 24;57(6):1549–62. doi: 10.1161/STROKEAHA.125.053989 (PMC13196860; doi:10.1161/STROKEAHA.125.053989)
Supplement: Supplementary file 1 [file str-57-1549-s001.pdf]

SUPPLEMENTAL MATERIAL

Figure S1 Unadjusted and adjusted cumulative mortality

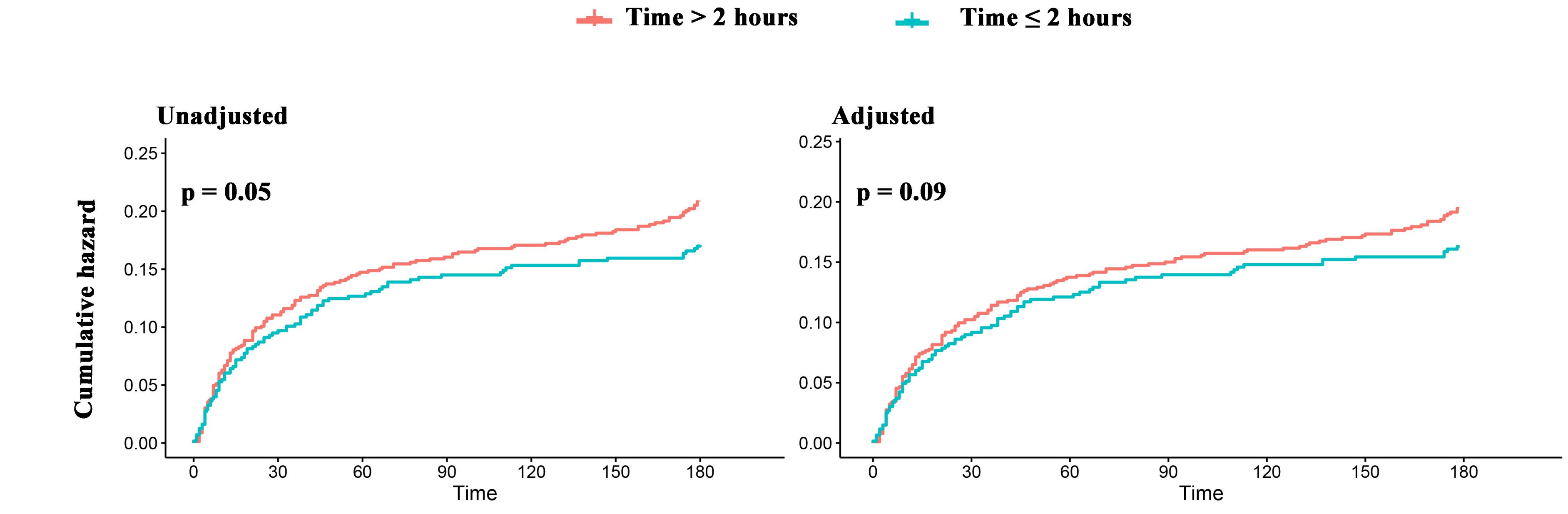

Unadjusted — Number at risk

Strata	0	30	60	90	120	150	180
Time > 2 hours	803	719	693	684	677	669	651
Time ≤ 2 hours	563	512	496	487	483	480	475

Adjusted — Number at risk

Strata	0	30	60	90	120	150	180
Time > 2 hours	802	718	692	683	676	668	650
Time ≤ 2 hours	562	511	495	486	482	479	474

Table S1. Subgroup analysis by location of hemorrhage and type of decompressive surgery

Outcome	Location of hemorrhage subgroups	OR [a] (95% CI)	*P* for interaction	Type of decompressive surgery subgroups	OR [a] (95% CI)	*P* for interaction
**Primary outcome**						
Death at 6 months [b]			0.91			0.31
	Cortical	0.65(0.27-1.53)		Craniotomy	0.74(0.53-1.03)	
	Deep	0.83(0.63-1.10)		Minimally invasive surgery	0.94(0.65-1.35)	
	Brainstem/Cerebellum	0.77(0.35-1.69)				
**Secondary outcomes**						
mRS shift [c]			0.46			0.31
	Cortical	0.84(0.43-1.63)		Craniotomy	0.68(0.52-0.89)	
	Deep	0.68(0.54-0.85)		Minimally invasive surgery	0.86(0.63-1.16)	
	Brainstem/Cerebellum	0.82(0.41-1.62)				
mRS score 4-6 [d]			0.68			0.43
	Cortical	0.75(0.34-1.66)		Craniotomy	0.67(0.49-0.92)	
	Deep	0.66(0.50-0.87)		Minimally invasive surgery	0.82(0.56-1.19)	
	Brainstem/Cerebellum	0.76(0.32-1.81)				
Residence at home at 6 months [d]			0.13			0.73
	Cortical	0.61(0.26-1.45)		Craniotomy	1.03(0.73-1.44)	
	Deep	1.22(0.91-1.62)		Minimally invasive surgery	1.18(0.79-1.74)	
	Brainstem/Cerebellum	0.93(0.35-2.46)				
Major disability at 6 months mRS:3-5 [d]			0.08			0.63
	Cortical	1.49(0.60-3.69)		Craniotomy	0.86(0.58-1.27)	
	Deep	0.74(0.53-1.03)		Minimally invasive surgery	0.97(0.64-1.47)	
	Brainstem/Cerebellum	1.34(0.53-3.40)				
Hematoma expansion [d]			0.98			0.35
	Cortical	0.82(0.26-2.66)		Craniotomy	0.85(0.53-1.38)	
	Deep	0.91(0.61-1.36)		Minimally invasive surgery	1.19(0.79-1.79)	
	Brainstem/Cerebellum	0.79(0.12-5.40)				
Death at day 7 [d]			0.18			0.20
	Cortical	0.55(0.04-7.27)		Craniotomy	0.54(0.21-1.40)	
	Deep	0.87(0.41-1.87)		Minimally invasive surgery	1.28(0.45-3.62)	
	Brainstem/Cerebellum	-				
Neurological deterioration [d]			0.86			0.42
	Cortical	0.67(0.18-2.50)		Craniotomy	0.52(0.31-0.86)	
	Deep	0.60(0.41-0.89)		Minimally invasive surgery	0.73(0.43-1.22)	
	Brainstem/Cerebellum	0.55(0.10-3.03)				
EQ-5D-3L, mobility [c]			0.28			0.40
	Cortical	1.02(0.45-2.30)		Craniotomy	0.72(0.53-0.99)	
	Deep	0.71(0.54-0.93)		Minimally invasive surgery	0.88(0.60-1.29)	
	Brainstem/Cerebellum	0.86(0.35-2.13)				
EQ-5D-3L, anxiety or depression [c]			0.30			**0.04**
	Cortical	1.20(0.49-2.93)		Craniotomy	1.19(0.82-1.72)	
	Deep	0.82(0.59-1.13)		Minimally invasive surgery	0.67(0.42-1.05)	
	Brainstem/Cerebellum	1.41(0.49-4.02)				
EQ-5D-3L, pain or discomfort [c]			0.95			0.49
	Cortical	0.62(0.26-1.46)		Craniotomy	0.77(0.54-1.11)	
	Deep	0.66(0.48-0.91)		Minimally invasive surgery	0.65(0.42-1.02)	
	Brainstem/Cerebellum	0.70(0.24-2.16)				
EQ-5D-3L, self-care [c]			0.13			0.57
	Cortical	1.15(0.52-2.56)		Craniotomy	0.80(0.59-1.09)	
	Deep	0.73(0.56-0.96)		Minimally invasive surgery	0.90(0.63-1.30)	
	Brainstem/Cerebellum	0.87(0.35-2.14)				
EQ-5D-3L, usual activities [c]			0.74			0.07
	Cortical	0.78(0.37-1.70)		Craniotomy	0.67(0.49-0.91)	
	Deep	0.75(0.59-0.99)		Minimally invasive surgery	1.03(0.72-1.49)	
	Brainstem/Cerebellum	0.90(0.40-2.04)				
EQ-5D-3L, visual analogue scale (log transform) [e]			0.22			0.25
	Cortical	-0.03(-0.28-0.23)		Craniotomy	0.04(-0.06-0.14)	
	Deep	0.12(0.02-0.21)		Minimally invasive surgery	0.14(0.00-0.28)	
	Brainstem/Cerebellum	0.09(-0.10-0.27)				
EQ-5D-3L, utility score [e]			0.72			0.27
	Cortical	0.05(-0.07-0.17)		Craniotomy	0.07(0.02-0.11)	
	Deep	0.06(0.02-0.10)		Minimally invasive surgery	0.03(-0.03-0.08)	
	Brainstem/Cerebellum	0.08(-0.05-0.21)				
All serious adverse events [f, d]			0.75			0.46
	Cortical	0.71(0.30-1.68)		Craniotomy	0.69(0.49-0.97)	
	Deep	0.73(0.55-0.97)		Minimally invasive surgery	0.83(0.56-1.22)	
	Brainstem/Cerebellum	0.52(0.21-1.25)				

Abbreviations: mRS, modified rankin scale; NIHSS, national institutes of health stroke scale; EQ-5D-3L, EuroQoL group 5-dimension self-report questionnaire; OR: odds ratio; CI: confidence interval.

Hematoma expansion defined as an increase of >6 mL or a growth of >33% at 24 hours.

Neurological deterioration: defined as an increase of ≥4 points in the NIHSS score at 7 days compared with baseline.

Minimally invasive surgery included endoscopy, aspiration, intraventricular drainage, catheterisation with/without lysis, and other type.

[a]OR for risks of outcome events for participants who achieved the blood pressure target within 2 hours compared to those who did not reach the target within 2 hours, adjusted for age, sex, GCS on admission and randomized treatment group.

[b]The risk of death at 6 months is represented as the hazard ratio, as the results were obtained using the Cox proportional regression hazards model.

[c]The risk of outcome events were estimated using an ordinal logistic regression model.

[d]The risk of outcome events were estimated using a logistic regression model.

[e]The mean differences in the visual analogue scale (log transform) and utility score were estimated using a linear regression model.

[f]Serious adverse events were defined as events that resulted in death, were life-threatening, required inpatient hospitalization or prolongation of an existing hospitalization, resulted in persistent or significant disability or incapacity, involved a congenital anomaly or birth defect, or were considered other medically important events; a patient could experience more than one SAE.

Table S2. Multivariable Models and Risk of Outcomes in Time to Reaching Target.

Outcome	Adjusted OR [a] (95% CI)	P Value
**Primary outcome**		
Death at 6 months [b]		
**>2h**	reference	
**≤2h**	0.90(0.70-1.17)	0.44
**Secondary outcomes**		
mRS shift [c]		
**>2h**	reference	
**≤2h**	0.80(0.65-0.98)	**0.038**
mRS score 4-6 [d]		
**>2h**	reference	
**≤2h**	0.78(0.60-1.01)	**0.059**
Residence at home at 6 months [d]		
**>2h**	reference	
**≤2h**	1.05(0.80-1.38)	0.73
Major disability at 6 months mRS:3-5 [d]		
**>2h**	reference	
**≤2h**	0.97(0.72-1.32)	0.86
Hematoma expansion [d]		
**>2h**	reference	
**≤2h**	1.03(0.69-1.54)	0.87
Death at day 7 [d]		
**>2h**	reference	
**≤2h**	0.83(0.40-1.73)	0.62
Neurological deterioration [d]		
**>2h**	reference	
**≤2h**	0.71 (0.49-1.03)	0.074
EQ-5D-3L, mobility [c]		
**>2h**	reference	
**≤2h**	0.83(0.64-1.07)	0.16
EQ-5D-3L, anxiety or depression [c]		
**>2h**	reference	
**≤2h**	0.87(0.65-1.18)	0.38
EQ-5D-3L, pain or discomfort [c]		
**>2h**	reference	
**≤2h**	0.66(0.49-0.89)	**0.0069**
EQ-5D-3L, self-care [c]		
**>2h**	reference	
**≤2h**	0.88(0.69-1.13)	0.32
EQ-5D-3L, usual activities [c]		
**>2h**	reference	
**≤2h**	0.83(0.64-1.07)	0.14
EQ-5D-3L, visual analogue scale (log transform) [e]		
**>2h**	reference	
**≤2h**	0.09(0.01-0.17)	**0.045**
EQ-5D-3L, utility score [e]		
**>2h**	reference	
**≤2h**	0.04(0.01-0.08)	**0.025**
All serious adverse events [f, d]		
**>2h**	reference	
**≤2h**	0.80(0.61-1.04)	0.10

Abbreviations: mRS, modified rankin scale; NIHSS, national institutes of health stroke scale; EQ-5D-3L, EuroQoL group 5-dimension self-report questionnaire; SBP, systolic blood pressure; DBP, diastolic blood pressure; OR: odds ratio; CI: confidence interval.

Hematoma expansion defined as an increase of >6 mL or a growth of >33% at 24 hours.

Neurological deterioration: defined as an increase of ≥4 points in the NIHSS score at 7 days compared with baseline.

[a]OR for risks of outcome events for participants who achieved the blood pressure target within 2 hours compared to those who did not reach the target within 2 hours, adjusted for age, sex, GCS on admission, baseline hematoma volume, presence or absence of IVH, SBP, DBP, premorbid functional status, baseline glucose level and randomized treatment group.

[b]The risk of death at 6 months is represented as the hazard ratio, as the results were obtained using the Cox proportional regression hazards model.

[c]The risk of outcome events were estimated using an ordinal logistic regression model.

[d]The risk of outcome events were estimated using a logistic regression model.

[e]The mean differences in the visual analogue scale (log transform) and utility score were estimated using a linear regression model.

[f]Nonfatal serious adverse events included those that were life threatening event, required inpatient hospitalization or prolongation of an existing hospitalization, or resulted in persistent or significant disability or incapacity.
